# Overexpression of sesame polyketide synthase A leads to abnormal pollen development in *Arabidopsis*

**DOI:** 10.1186/s12870-022-03551-7

**Published:** 2022-04-02

**Authors:** Tianyu Li, Yuanxiao Yang, Hongyan Liu, Senouwa Segla Koffi Dossou, Fang Zhou, Ting Zhou, Yingzhong Zhao

**Affiliations:** Key Laboratory of Biology and Genetic Improvement of Oil Crops, Ministry of Agriculture and Rural Affairs, Oil Crops Research Institute of the Chinese Academy of Agricultural Sciences, Wuhan, 430062 China

**Keywords:** Sesame, Male sterility, Exine, Sporopollenin, *SiPKSA*

## Abstract

**Background:**

Sesame is a great reservoir of bioactive constituents and unique antioxidant components. It is widely used for its nutritional and medicinal value. The expanding demand for sesame seeds is putting pressure on sesame breeders to develop high-yielding varieties. A hybrid breeding strategy based on male sterility is one of the most effective ways to increase the crop yield. To date, little is known about the genes and mechanism underlying sesame male fertility. Therefore, studies are being conducted to identify and functionally characterize key candidate genes involved in sesame pollen development. Polyketide synthases (PKSs) are critical enzymes involved in the biosynthesis of sporopollenin, the primary component of pollen exine. Their *in planta* functions are being investigated for applications in crop breeding.

**Results:**

In this study, we cloned the sesame POLYKETIDE SYNTHASE A (*SiPKSA*) and examined its function in male sterility. *SiPKSA* was specifically expressed in sesame flower buds, and its expression was significantly higher in sterile sesame anthers than in fertile anthers during the tetrad and microspore development stages. Furthermore, overexpression of *SiPKSA* in *Arabidopsis* caused male sterility in transgenic plants. Ultrastructural observation showed that the pollen grains of *SiPKSA*-overexpressing plants contained few cytoplasmic inclusions and exhibited an abnormal pollen wall structure, with a thicker exine layer compared to the wild type. In agreement with this, the expression of a set of sporopollenin biosynthesis-related genes and the contents of their fatty acids and phenolics were significantly altered in anthers of *SiPKSA*-overexpressing plants compared with wild type during anther development.

**Conclusion:**

These findings highlighted that overexpression of *SiPKSA* in *Arabidopsis* might cause male sterility through defective pollen wall formation. Moreover, they suggested that *SiPKSA* modulates vibrant pollen development via sporopollenin biosynthesis, and a defect in its regulation may induce male sterility. Therefore, genetic manipulation of *SiPKSA* might promote hybrid breeding in sesame and other crop species.

**Supplementary Information:**

The online version contains supplementary material available at 10.1186/s12870-022-03551-7.

## Background

Sesame (*Sesamum indicum* L.) is one of the most ancient oil crops and is widely used for its high-quality products. Its seeds are a great reservoir of oil, protein, unsaturated fatty acids, lignans, vitamins, and minerals [1, 2]. Moreover, many studies have revealed that sesame lignans possess various health-promoting abilities against lifestyle diseases [3–6]. Accordingly, the sesame seed market is expanding, placing great pressure on sesame breeders to develop environmentally stable and high-yielding varieties. A hybrid breeding strategy based on sterile male lines is one of the most reliable techniques to increase crop yield. Therefore, deciphering the molecular mechanism of fertility-related genes in sesame might provide genetic resources and new ideas for the crop improvement.

Pollen is a critical organ in angiosperm plant reproduction that facilitates pollination and fertilization [7]. Accordingly, its formation represents a key step of sexual reproduction in flowering plants. Male reproductive organ development is a complex biological process that consists of various biosynthesis events, among which pollen wall formation is essential for pollen viability and fertility [7, 8]. Any defects during pollen wall formation may lead to abnormal pollen and male sterility [9].

The pollen wall is an important structure of the outer surface of pollen. It comprises three layers: the pollen coat, the outer exine, and the inner intine [10]. The exine, a lipidic structure layer, is divided into two sublayers: an outer sculptured layer (the sexine) and a plain inner layer (the nexine). The sexine is constructed of rod-like structures called baculum, which are covered by a roof (of different shapes) called tectum [11]. Pollen exine patterning mainly includes three developmental events: primexine formation, callose wall formation, and sporopollenin synthesis [7, 10, 12]. Sporopollenin, a complex of fatty acids and phenolic compounds, represents the principal component that constitutes the pollen exine architecture [13].

Studies in *Arabidopsis* revealed that sporopollenin biosynthesis occurs in tapetal secretory cells after the temporary callose wall surrounding tetrads is degraded and primexine is deposited [14]. To date, several sporopolonin biosynthesis-related genes have been identified, such as ACYL-COA SYNTHETASE5 (*ACOS5*), Cytochrome P450 A2 (*CYP703A2*), Cytochrome P450 B1 (*CYP704B1*), MALE STERILITY2 (*MS2*), TETRAKETIDE a-PYRONE REDUCTASE1 (*TKPR1*), TETRAKETIDE a-PYRONE REDUCTASE 2 (*TKPR2*), POLYKETIDE SYNTHASEA (*PKSA*) and POLYKETIDE SYNTHASEB (*PKSB*) [15–21]. The sporopollenin biosynthesis pathway is conserved in land plant [7, 22–25]. Among the genes that contribute to vibrant and fertile pollen formation, polyketide synthases are critical [15, 16]. They catalyze the condensation of acyl-CoA molecules into hydroxyalkyl α-pyrone, the precursor of sporopollenin [7]. The *Arabidopsis pksa/lap6* or *pksb/lap5* mutant produces abnormal pollen exine, while the double mutant develops collapsed pollen without exine deposition and is male sterile [15, 16]. Kim et al. demonstrated that *Arabidopsis POLYKETIDE SYNTHASE A/PKSA* and *PKSB* (also known as *LAP6* and *LAP5*, respectively) interact continuously with other pollen exine biosynthesis genes during anther development [15]. The integration of increasing genomic information and advanced genetic tools facilitates the transfer of information from *Arabidopsis* to other plants, including rice and rapeseed, in which orthologs of *PKSA* have been identified and characterized [26–28]. Manipulation of male fertility has been shown to be an effective method for hybrid breeding and increasing crop yields [29]. Therefore, genetic manipunation of *PKSA* gene might enhance hybrid breeding.

In sesame, although early studies reported strong heterosis [30, 31], few nuclear male sterile lines have been used in hybrid breeding [32, 33]. Studies on sesame male sterility were limited to the identification of two genes, *Sesamum indicum male sterility 1* (*SiMs1*) [34] and *Sesamum indicum cell wall invertase 1* (*Sicwinv1*) [35]. In the present study, sesame *polyketide synthase A* (*SiPKSA*) was cloned and functionally characterized to be involved in male reproduction. Our results suggest that *SiPKSA* might be involved in sporopollenin biosynthesis to affect pollen and pollen wall formation and it is a potential candidate gene for the improvement of sesame seed yield through hybrid breeding.

## Results

### Cloning and sequence analysis of *SiPKSA*

Sesame polyketide synthase, the ortholog of *Arabidopsis PKSA*, designated *SiPKSA* (*Sesamum indicum* Polyketide Synthase A), was cloned using the cDNA of sesame anthers at the tetrad stage as the template. The open reading frame (ORF) of *SiPKSA* is 1191 bp, encoding 396 amino acids, with an isoelectric point of 6.41, and a molecular weight of 43.6 kDa.

**Fig. 1:**
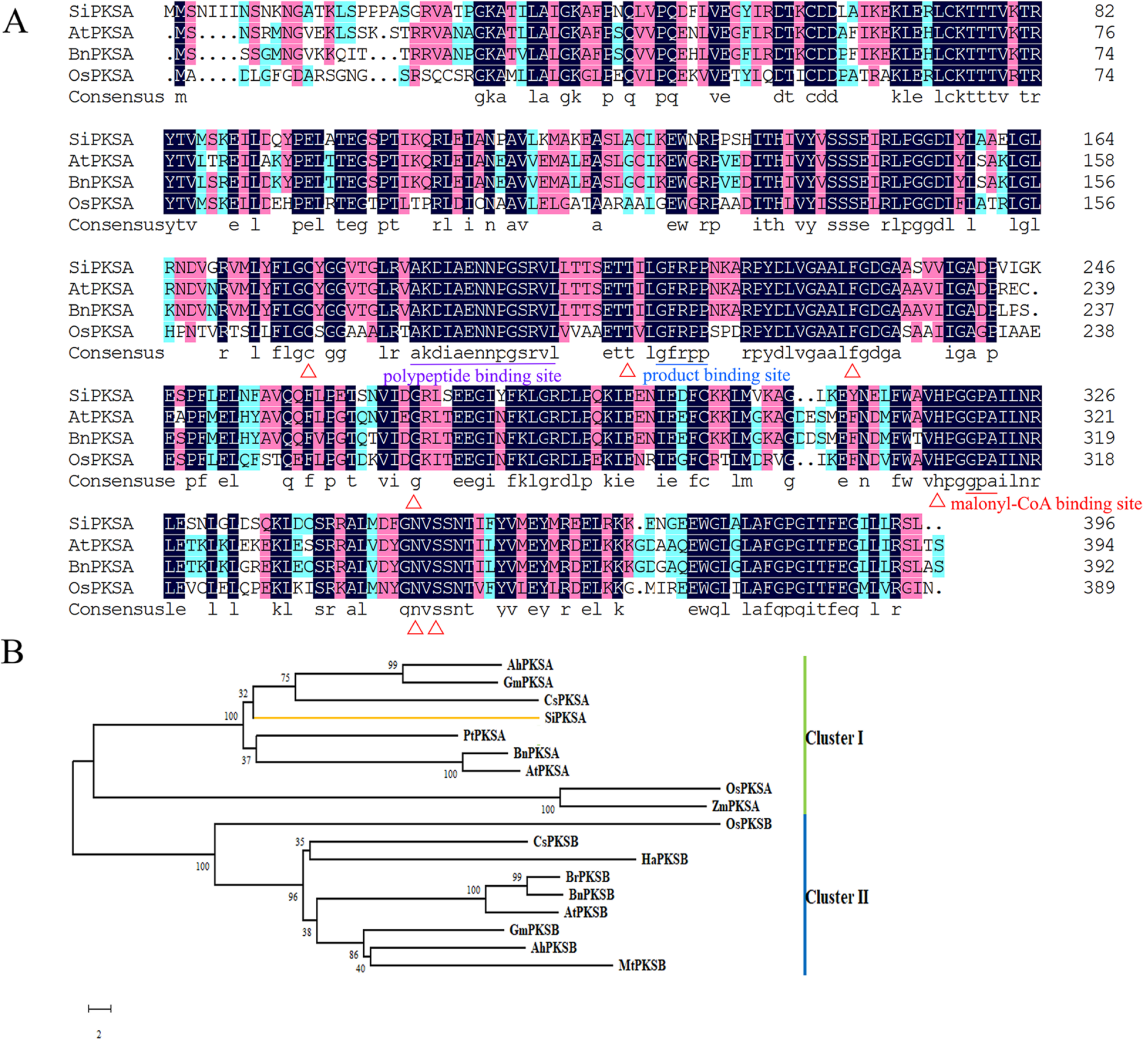
Multiple sequence alignment and phylogenetic analysis of *SiPKSA* with plant-specific type III polyketide synthases (PKSs) in other species. **A** Protein sequence alignment of *SiPKSA* with anther developmental-related PKSA from other crops. The conserved sites are underlined, and the amino acid residues are framed. *AtPKSA* (At1g02050), *BnPKSA* (XP_013701181.1), *OsPKSA* (NP_001064891.1). **B** Phylogenetic analysis of *SiPKSA* with PKSs from other species. *Sesame indium* (Si); *Arabidopsis thaliana* (At); *Zea mays* (Zm); *Glycine max* (Gm); *Populus trichocarpa* (Pt); *Oryza sativa* (Os); *Brassica napus* (Bn); *Medicago truncatula* (Mt); *Citrus sinensis* (Cs); *Helianthus annuus* (Ha); *Arachis hypogaea* (Ah); *Brassica rapa* (Br). The bootstrap values are shown at the nodes

Multiple sequence alignment of the SiPKSA protein with other PKSA proteins reported in *Arabidopsis* (*Arabidopsis thaliana*), rice (*Oryza sativa*), and rape (*Brassica napus*) showed that the SiPKSA protein contains similar domains as polyketide synthase A in other species, including the typical chalcone/stilbene synthase N-terminal domain (25–242 bp), C-terminal domain (252–396 bp) and various conserved active sites, such as polypeptide binding site, malonyl-CoA binding site, and product binding site, as well as the catalytic triad Cys-His-Asn, which is common and important in the PKS family (Fig. 1A). The phylogenetic analysis of SiPKSA and polyketide synthases in other species indicated that SiPKSA is closer to PKSA in other species (Fig. 1B), confirming that *SiPKSA* encodes a polyketide synthase A protein.

In addition, the promoter region of *SiPKSA* was analyzed using the PLACE and PLANTCARE databases. We found that a large number of formal elements were annotated as MYB recognition sites, light-responsive elements, and auxin-responsive elements. Notably, multiple copies of AGAAA, which is considered as an anther- and/or pollen-specific cis-regulatory motif, are predicted in the promoter region of *SiPKSA*. Other motifs, such as Q-elements and GTGA motifs related to pollen development were also predicted in the promoter region (Table S2), indicating that *SiPKSA* might be involved in pollen development in sesame.

### *SiPKSA* is predominantly expressed in sesame reproductive tissue

**Fig. 2:**
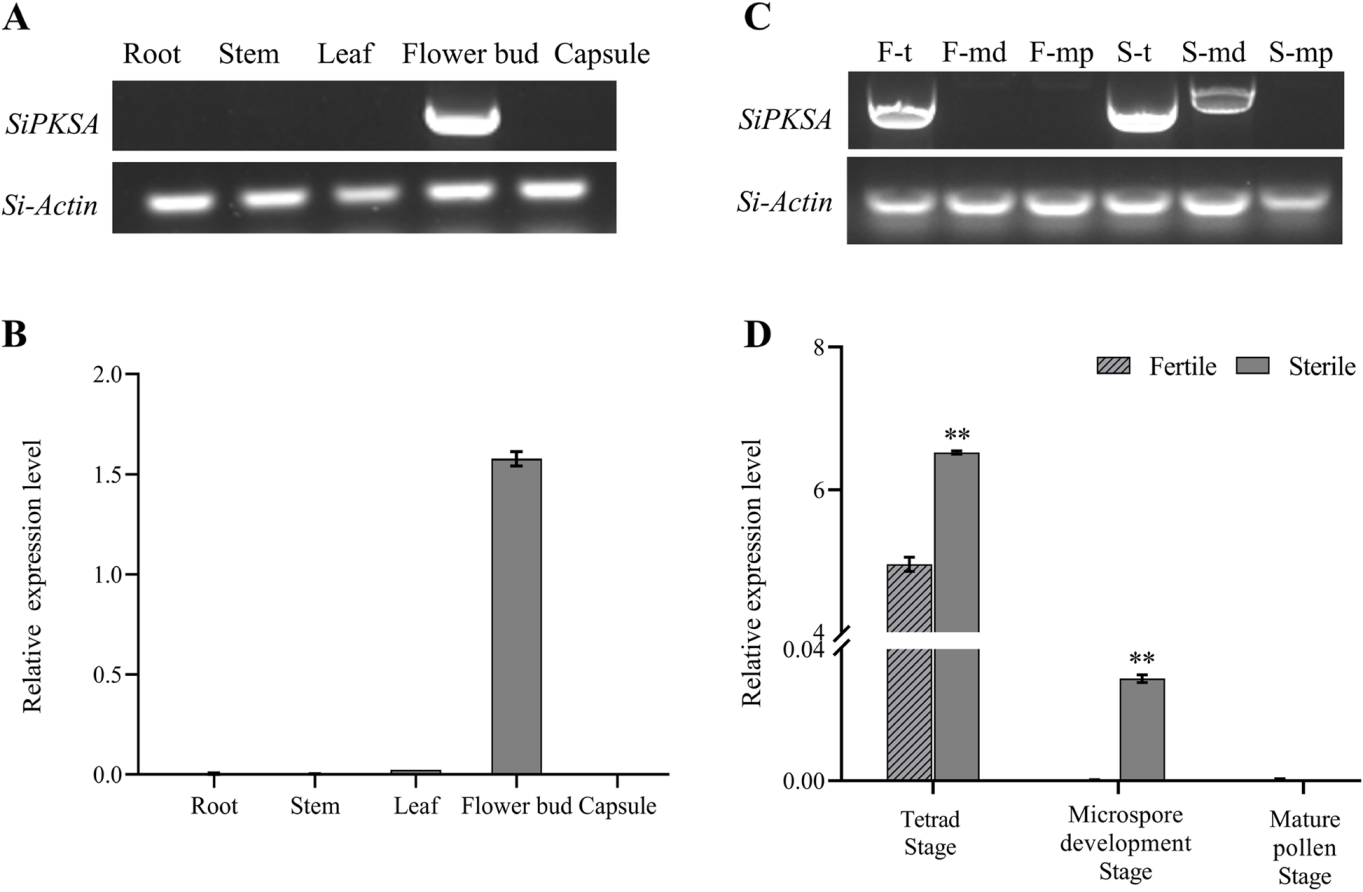
Expression analysis of *SiPKSA* in sesame. **A-B** The expression profile of *SiPKSA* in different sesame tissues by RT-PCR (A) and qRT-PCR analysis (**B**). **C-D** RT-PCR (**C**) and qRT-PCR analysis (**D**) of the expression of *SiPKSA* in sesame fertile and sterile anthers at different developmental stages. S-t, S-md, and S-mp represent the tetrad stage, microspore development stage, and mature pollen stage of the sterile anthers, respectively. F-t, F-md, and F-mp represent the tetrad stage, microspore development stage, and mature pollen stage of the fertile anthers, respectively. Si*-*Actin (SIN_1009011) was used as the internal reference control. Three biological replicates were conducted and the error bars represent ± SD. Statistically significant differences were assessed using Student’s t-test (**p < 0.01 is considred a highly significantly different)

The expression pattern of *SiPKSA* in different organs of sesame was analyzed first to determine whether *SiPKSA* is tissue-specific. The results revealed that *SiPKSA* is predominantly expressed in flower buds (Fig. 2A and B), indicating that its function in sesame might be related to plant reproduction. Furthermore, to determine the potential role of *SiPKSA* in sesame male sterility, we compared the expression pattern of *SiPKSA* in sterile and fertile sesame anthers at different developmental stages. We observed that the expression of *SiPKSA* was significantly higher in sterile sesame anthers at the tetrad stage and microspore development stage than in fertile anthers (Fig. 2C and D), suggesting that high expression of *SiPKSA* might induce male sterility in sesame. Thus, normal activity and levels of *SiPKSA* might be required for sesame male fertility.

### Overexpressing *SiPKSA* in *Arabidopsis* caused male sterility in transgenic plants

**Fig. 3:**
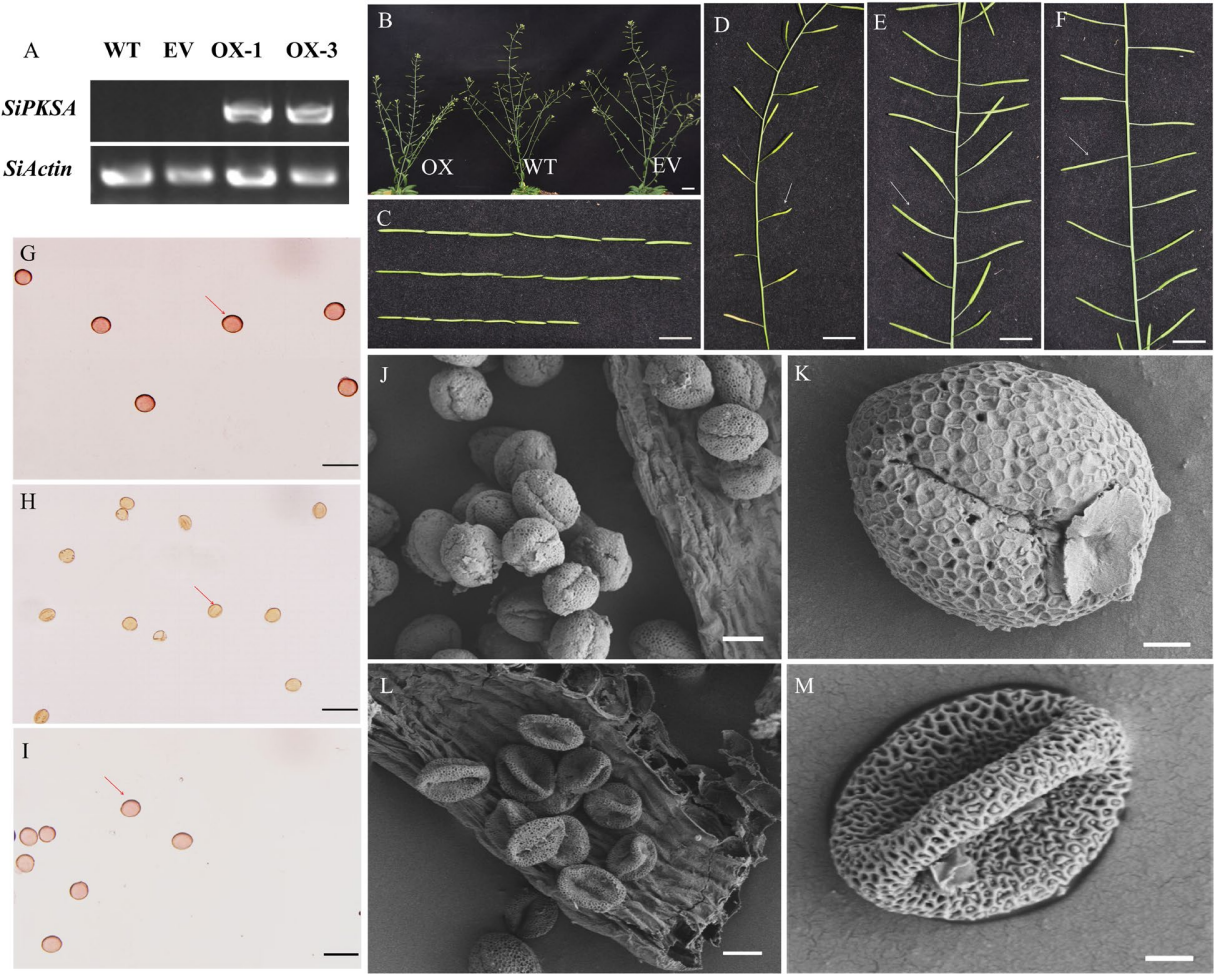
Ectopic expression of *SiPKSA* in *Arabidopsis* caused male sterility of transgenic plants. **A** RT-PCR analysis of *SiPKSA* in *SiPKSA*-overexpressing lines (OX-1, OX-3), wild-type (WT) and plants transformed with empty vector (EV). At-Actin7 (At5g09810) was used as an internal standard. **B** The phenotype of 8-week-old *SiPKSA*-overexpressing *Arabidopsis* plants. The scale bar represents 2 cm. **C** Comparison of the length of the siliques of *SiPKSA*-overexpressing *Arabidopsis* plants (below), WT (middle), and EV (above) (**D-E**) Magnified image of *SiPKSA*-overexpressing plant (**D**), WT plant (**E**), and EV (**F**). The white arrow indicated the siliques of the plants. The scale bars represent 1 cm. (G-I) Detection of pollen vigor of WT (**G**), *SiPKSA-*overexpressing plants (**H**) and EVs (**I**) with 0.5% acetocarmine reagent. The red arrow shows the stained pollen. The pollen grains of WT and EV were dyed red, while the pollen grains of *SiPKSA*-overexpressing plant were not colored. **J**, **K** The phenotype of pollen grains of WT under SEM. **L**, **M**. The phenotype of pollen grains of *SiPKSA*-overexpressing plants under SEM. Scale bars represent 100 μm (**G-I**), 10 μm (**J**, **L**) and 2 μm (**K**, **M**)

To further assess the in *planta* function of *SiPKSA* and investigate the effects of its high expression during anther development, we engineered *Arabidopsis* plants to overexpress the *SiPKSA* gene under the 35 s promoter. Several transgenic lines were harvested, among which we selected two independent homozygous lines in the T3 generation for phenotypic and functional analyses. The expression of *SiPKSA* was highly induced in the two independent homozygous lines compared to the wild type plants (WT) and the plants transformed with empty vector (EV) (Fig. 3A). The expression level of the *Arabidopsis* homologous gene *AtPKSA* was also detected. The results showed that there were no significant differences between *SiPKSA*-overexpressing plants, wild-type plants, or plants transformed with empty vector in terms of the expression level of *AtPKSA* both in seedlings and anthers at different developmental stages (Fig. S1A-C), suggesting that the phenotype of transgenic plants in the present study was caused by *SiPKSA.*

As shown in Fig. 3B, no significant phenotypic difference was observed between the wild-type (WT) plants, the plants transformed with empty vector (EV), and the *SiPKSA*-overexpressing plants, except for the silique size. The siliques of *SiPKSA*-overexpressing plants were flat and short compared to those of the wild type (WT) and the plants transformed with the empty vector (EV) (Fig. 3C-E).

To enhance the contrast of the pollen grains under the microscope, acetocarmine solution also known as a nuclear and chromosomal fixation and staining agent, was used for staining to investigate the pollen phenotype. Microscopic observations showed that the pollen grains of WT and EV were plump and darkly stained (Fig. 3F and H), indicating high pollen viability. In contrast, the pollen grains of *SiPKSA*-overexpressing plants were uncolored and small, indicating poor vigor (Fig. 3G).

To verify the above observations, we scanned the pollen using scanning electron microscopy (SEM). The results confirmed that the WT pollen grains were normal and those of the *SiPKSA*-overexpressing plants were abnormal. The WT pollen grains were full with a typical sculptured surface (Fig. 3I and J), while those of the *SiPKSA-*overexpressing plants were shrunken and collapsed (Fig. 3K and L). These results indicated that *SiPKSA*-overexpressing plants were male sterile.

### Abnormal pollen and pollen wall development in *SiPKSA-*overexpressing *Arabidopsis* plants

**Fig. 4:**
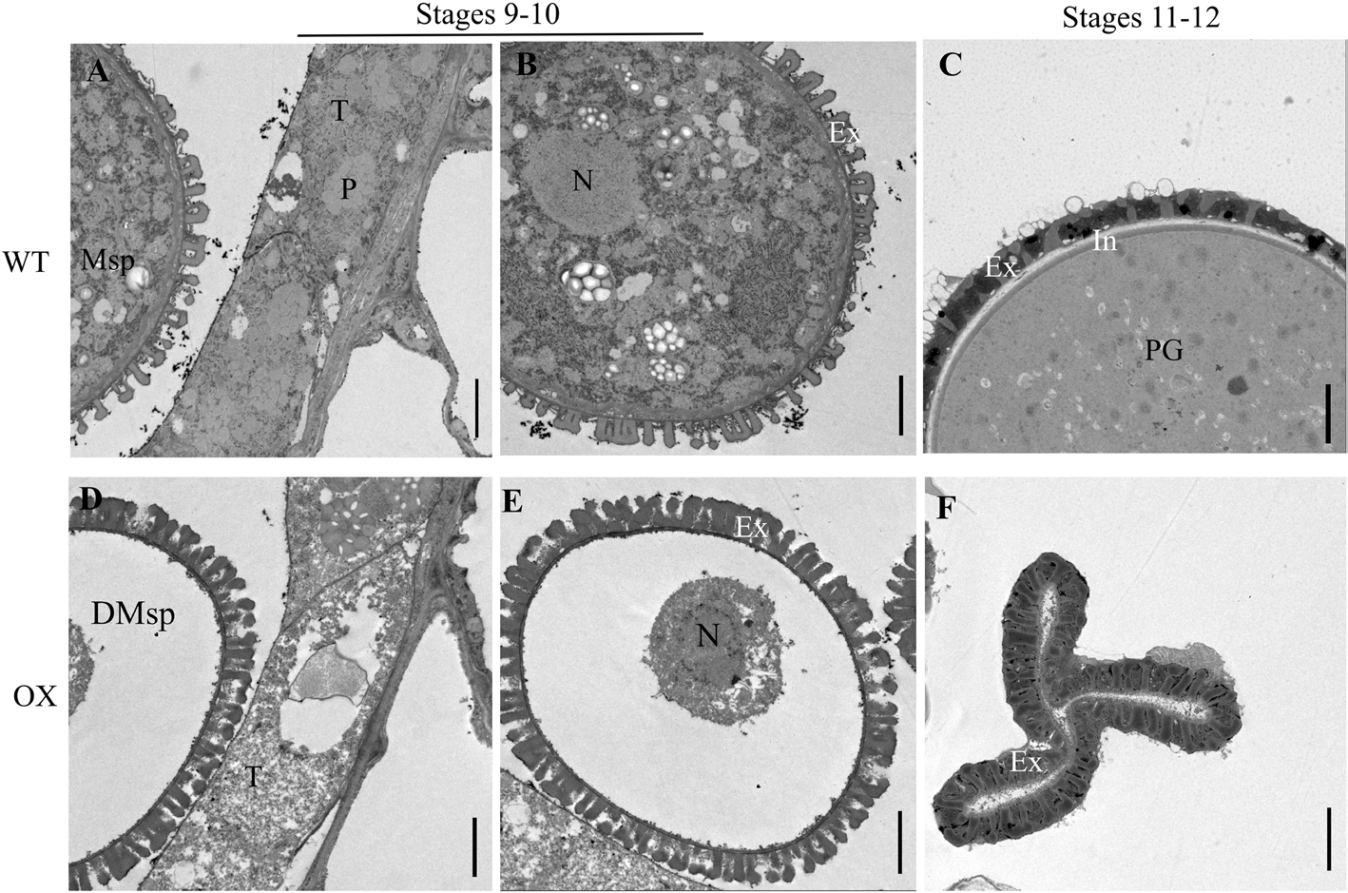
Transmission electron micrographs (TEM) observation of the ultrastructure of microspores and tapetum development of wild-type and *SiPKSA*-overexpressing plants at different anther developmental stages. **A**, **C** Tapetal cells of the WT (**A**) and *SiPKSA*-overexpressing plants during stages 9–10 (**C**). **B**, **D** Microspores of the WT (**B**) and *SiPKSA*-overexpressing plants during stages 9–10 (**D**). (**E**, **F**) Pollen walls of WT (**E**) and *SiPKSA*-overexpressing plants (**F**) at stages 11–12. Msp, microspore; DMsp, degenerated microspore; T, tapetum; In, intine; Ex, exine; N, nucleus; P, plastid filled with plastoglobuli; PG, pollen grain. Scale bars are 2 μm

**Fig. 5:**
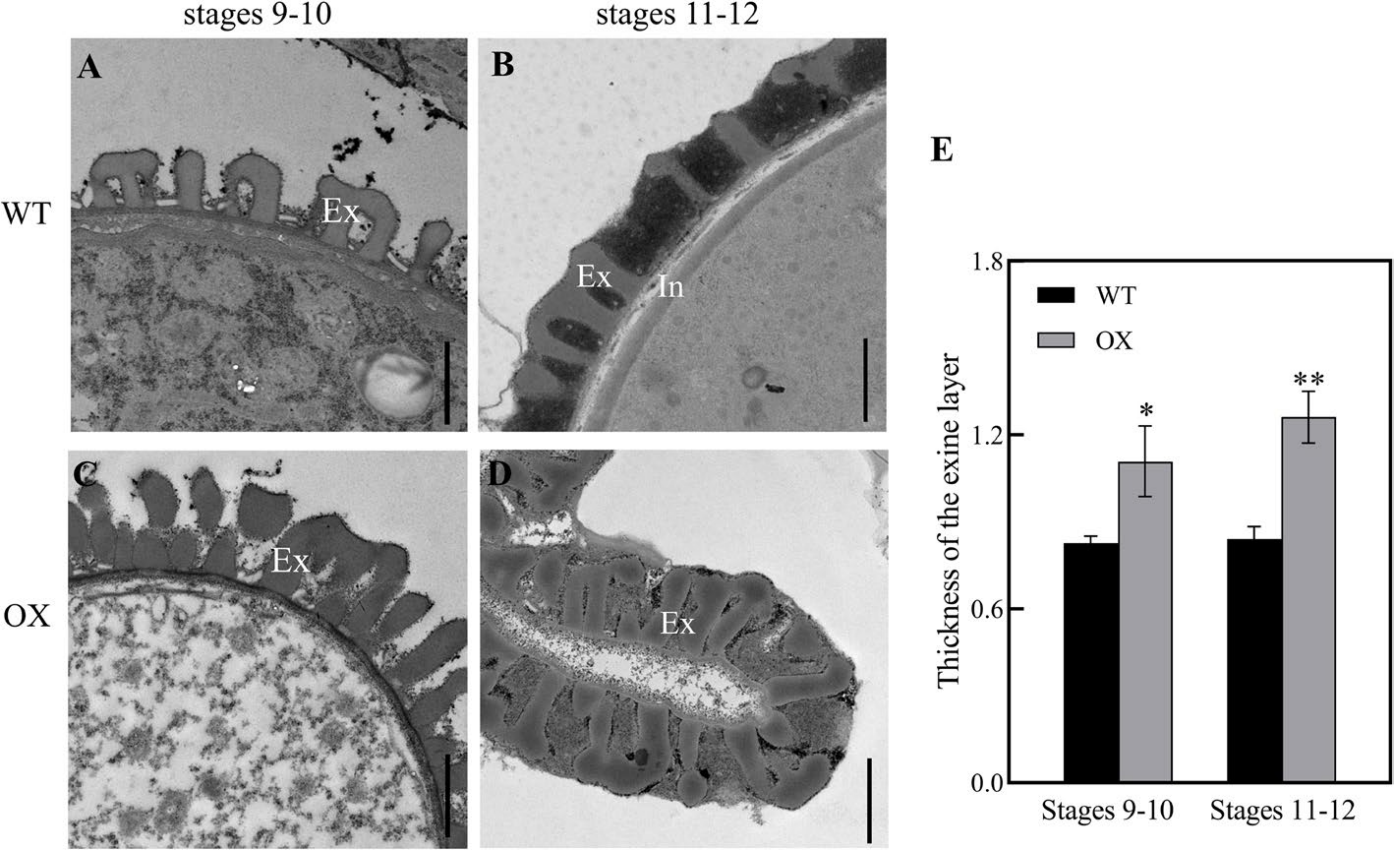
TEM observation of pollen walls from wild-type (WT) and *SiPKSA*-overexpressing plants at different anther developmental stages. **A**-**D** The ultrastructure of the pollen wall of WT and *SiPKSA*-overexpressing plants at anther developmental stages 9–10 and 11–12. **E** The thickness of the exine layer at stages 9–10 and 11–12. Ex, exine; In, intine. The scale bars are 1 μm. Three biological replicates were conducted, and the error bars indicate ± SD. The measurements were repeated ten times for each sample. Statistically significant differences were assessed using Student’s t-test (*p < 0.05 is considred a significantly different, **p < 0.01 is considred a highly significantly different)

According to the cytological events, *Arabidopsis* anther development is characterized into 14 stages, and the microspores differentiate into mature pollen during stages 9–12 [8]. We then conducted transmission electron microscopy (TEM) observations to further investigate the ultrastructure of the pollen and pollen walls during anther development. Our results showed that during stages 9–10, the tapetum of WT contained small vacuoles and abundant plastids filled with plastoglobuli (plastid lipoprotein particles), which can synthesize and store the carbohydrates and lipids needed for microspore development (Fig. 4A). The microspores of WT were rich in inclusions and embedded within the regular primary structure of the exine (Fig. 4B). However, in the tapetal cells of *SiPKSA*-overexpressing plants, large cavities and fewer cytoplasmic inclusions were observed (Fig. 4C). The microspores of *SiPKSA*-overexpressing plants were obviously abnormal with few cytoplasmic components, and compared with the WT, the sporopollenin particles on the pollen exine of the *SiPKSA*-overexpressing plants were abundant, disorderly and sparsely deposited on the microspore surface (Fig. 4D) During stages 11–12, the pollen of WT exhibited a typical normal pollen wall with obvious exine and intine layers (Fig. 4E). In contrast, the pollen grains of *SiPKSA*-overexpressing plants were seriously collapsed, with irregular exines and intines (Fig. 4F). Regarding the thickness of the pollen exine during stages 9–10 and 11–12, the results showed that the exine layer of *SiPKSA*-overexpressing plants was significantly thicker than that of the WT (Fig. 5A-E).

### Overexpression of *SiPKSA* altered the expression of sporopollenin biosynthesis-related genes and fatty acid metabolism

**Fig. 6:**
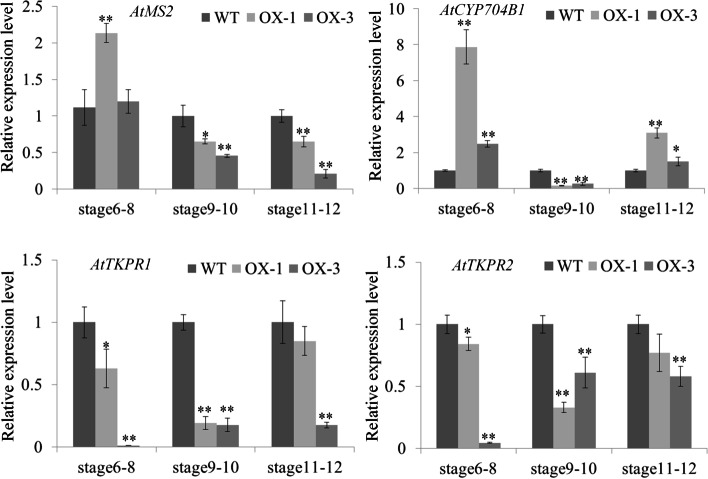
qRT-PCR analysis of the pollen development-related genes in the controls and *SiPKSA*-overexpressing plants. The expression of the pollen development-related genes are altered in anthers of *SiPKSA*-overexpressing plants compared with WT during anther development. Three biological replicates were performed, and the error bars represent ± SD. The expression was normalized to *AtActin7* (At5g09810). Statistically significant differences were assessed using Student’s t-test (*p < 0.05 is considred a significantly different, **p < 0.01 is considred a highly significantly different). *AtMS2* (At3g11980), *AtCYP704B1* (At1g69500), *AtTKPR1* (At4g35420), and *AtTKPR2* (At1g62940)

The main component of pollen exine is sporopollenin, therefore, the expression of several sporopollenin biosynthesis-related genes, including *AtMS2, AtCYP704B1, AtTKPR1,* and *AtTKPR2* was analyzed by qRT-PCR*.* Comparing to WT, the expression of *AtMS2* was up-regulated in anthers of *SiPKSA*-overexpressing plants during stages 6–8, and significantly down-regulated during stages 9–10 and 11–12. The expression of *AtCYP704B1* was significantly elevated in anthers of SiPKSA-overexpressing plants during stages 6–8, and dramatically reduced during stages 9–10, and significantly elevated during stages 11–12. The expression of *AtTKPR1* and *AtTKPR2* was remarkably down-regulated in anthers of *SiPKSA*-overexpressing plants during stages 6–8, 9–10, and 11–12 (Fig. 6 and Table S3). These results indicated that the expression of sporopollenin biosynthesis-related genes is influenced by the high expression of *SiPKSA* in transgenic *Arabidopsis*.

**Fig. 7:**
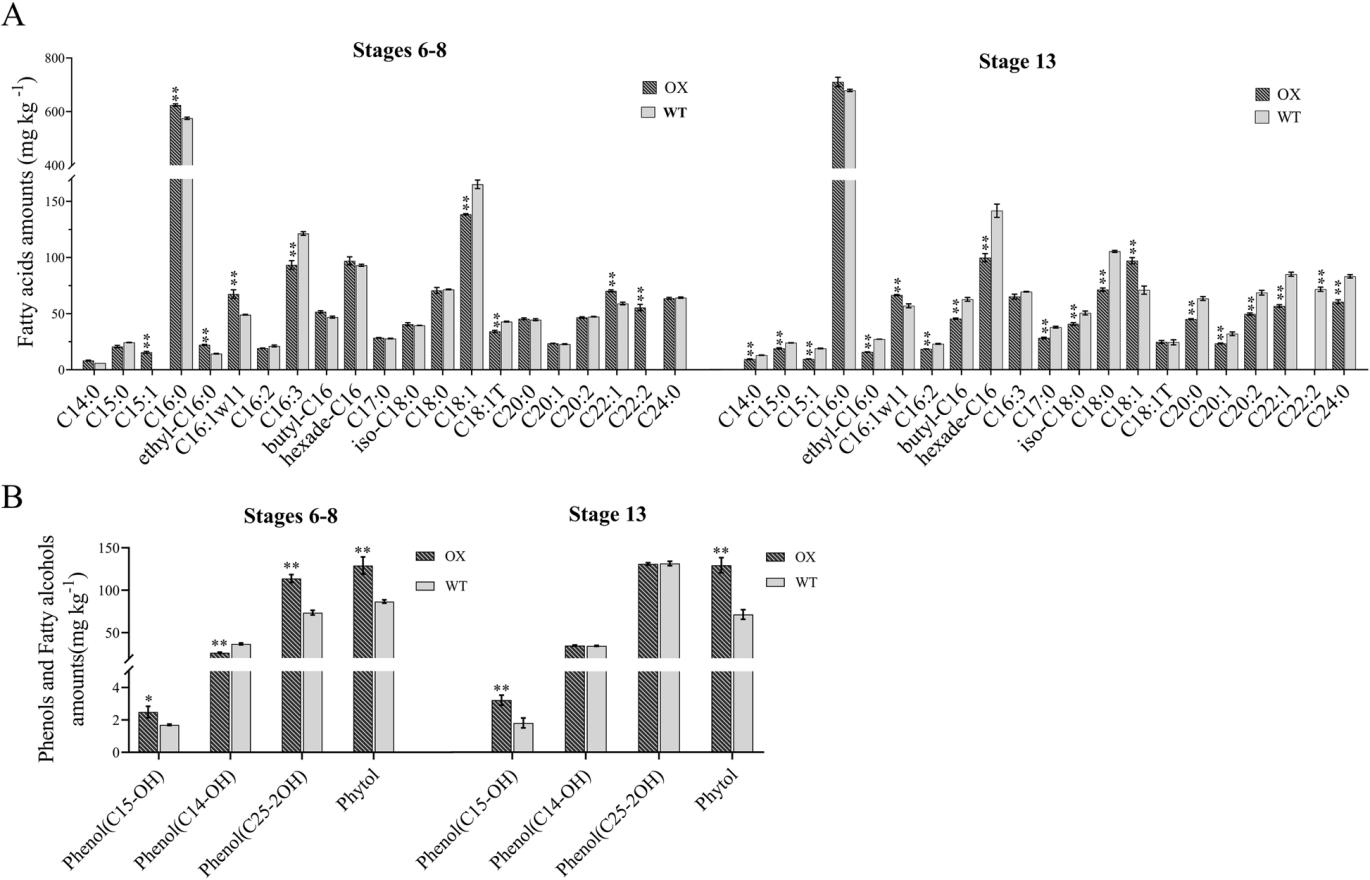
Measurement of fatty acids and their derivatives in anthers of *SiPKSA-*overexpressing plants and wild-type plants by GC–MS. **A** The content of fatty acids in anthers at stages 6–8 and 13. **B** The contents of phenols and fatty alcohols in anthers at stages 6–8 and 13. C12:0, Lauric acid methyl ester; C14:0, Methyl tetradecanoate; C15:0, Pentadecanoic acid methyl ester; C15:1, 9-Octadecenoic acid methyl ester; C16:0, Hexadecanoic acid methyl ester; ethyl-C16:0, 9-Hexadecenoic acid methyl ester; C16:1ω11, 11-Hexadecenoic acid methyl ester; C16:2, 7,10-Hexadecadienoic acid methyl ester; C16:3, 7,10,13-Hexadecatrienoic acid methyl ester; hexade-C16, Hexadecanedioic acid dimethyl ester; butyl-C16, butyl isobutyl phthalate; C17:0, Heptadecanoic acid methyl ester; C18:0, Methyl stearate; iso-C18:0, Methyl isostearate; C18:1, (Z)-9-Octadecenoic acid methyl ester; C18:1 T, 9,12,15-Octadecatrienoic acid ethyl ester; C20:0, Arachidic acid methyl ester; C20:1, cis-11-Eicosenoic acid methyl ester; C20:2, 11,13-Eicosadienoic acid methyl ester; C22:1, 13-Docosenoic acid methyl ester; C22:2, cis-13,16-Docasadienoic acid methyl ester; C24:0, Tetracosanoic acid methyl ester; Phenol(C15-OH), Butylated Hydroxytoluene; Phenol(C14-OH), 2,4-bis(1,1-dimethylethyl)-Phenol; Phenol(C25-2OH), 2,2′-methylenebis[6-(1,1-dimethylethyl)]-4-methyl-Phenol. Three biological replicates were performed and the error bars represent ± SD. Statistically significant differences were assessed using Student’s t-test (**p < 0.01 is considred a highly significantly different)

The fatty acids composition of the anthers at different developmental stages was further investigated by GC–MS analysis. The results showed that, in comparison with the WT, the content of several long-chain fatty acids was greatly affected in anthers of *SiPKSA*-overexpressing plants during the anther development. During stages 6–8, the contents of C15:1, C16:0, ethyl-C16, C16:1ω11, C22:1, and C22:2 were significantly increased, while the contents of C16:3, C18:1, and C18:1 T were significantly decreased. During stage 13, the content of most long-chain fatty acids decreased, while the content of C16:1ω11 and C18:1 increased (Fig. 7A). In addition, the content of three phenolic compoundswas significantly influenced. The C15-OH and C25-2OH contents significantly increased, while the C14-OH content decreased during stages 6–8 in *SiPKSA*-overexpressing plants (Fig. 7B). The fatty alcohol (Phytol) content greatly increased at both stages 6–8 and stage 13 in *SiPKSA*-overexpressing plants (Fig. 7B). These results indicated that normal fatty acid metabolism was affected in anthers of *SiPKSA*-overexpressing plants, consistent with the abnormal pollen and pollen wall development in the *SiPKSA*-overexpressing plants.

## Discussion

### *SiPKSA* is a typical chalcone synthase with a similar function as its homologs

Polyketide compounds are usually produced under the action of polyketide synthases (PKSs). PKSs can be divided into three types (I, II, and III) according to their protein structure [36, 37]. Type III PKSs, also known as the chalcone synthase (CHS) superfamily, are responsible for the biosynthesis of monocyclic or bicyclic aromatic polyketide compounds [38]. A series of type III PKSs with different functions have been characterized in plants, such as chalcone synthase (CHS), stilbene synthase (STS), acribone synthase (ACS), and 2-pyrone synthase (2-PS). These type III PKSs harbor a highly conserved catalytic active center (Cys164-His303-Asn336), and their active amino acid residues, generally Thr197, Phe215, Gly256, and Ser338, are located in the cavity of the active center [39]. However, the active site and amino acid residues of different functions of type III PKSs are slightly different to enable the production of diverse products [40].

In this study, multiple sequence alignment results showed that the SiPKSA protein possesses a typical chalcone synthase structure and conserved active catalytic centers with active amino acid residues as per the AtPKSA, BnPKSA, and OsPKSA/OsPKS1 proteins (Fig. 1A). Additionally, the phylogenetic analysis grouped the SiPKSA protein and PKSAs from other species (Fig. 1B). These findings indicate that SiPKSA is a type III PKSs and is homologous to other PKSAs. It has been reported that plant-specific CHS-like type III PKSs *LAP6/PKSA* and *LAP5/PKSB* are specifically expressed in anthers and are critical for proper pollen development [16]. *OsPKS1, a homolog of LAP6/PKSA*, also has a similar function [23].

In agreement with this finding, *SiPKSA* was expressed specifically in sesame flower buds (Fig. 2A-B). Overexpression of *SiPKSA* in Arabidopsis caused male sterility in transgenic plants. The pollen grains of *SiPKSA*-overexpressing plants were collapsed and embedded within a defective pollen wall (Fig. 4). These results suggest that *SiPKSA* plays a similar function as its homologs and is specifically involved in plant reproduction.

### *SiPKSA* is required for proper pollen exine formation and might be involved in sporopollenin biosynthesis

In Arabidopsis, the *pksa* mutant showed an incompletely male-sterile phenotype, with a thinner pollen exineand a shorter baculum and tectum compared to the wild-type plant [15]. In rice, the *OsPKS1* knockout mutant obtained by CRISPR-Cas9-mediated genome editing exhibited a complete male-sterile phenotype. Cytological observations revealed that the baculum of the rice *Ospks1* mutants was shorter, granular, and collapsed, with abnormal deposits [23]. A single-nucleotide polymorphism (SNP) mutation of the *OsPKS1* gene also showed the same phenotype of male sterility caused by defective pollen walls [24]. In addition, it has been reported that mutants of other sporopollenin biosynthesis-related genes, including *CYP704B1*, *MS2*, *TKPR1*, and *TKPR2*, exhibit defective or absent pollen exine [15–21]. These reports all showed that downregulation of these genes causes decreased sporopollenin biosynthesis, leading to lack of or much thinner pollen exine layers. However, the male sterility of the cotton Line 1335A is associated with upregulation of the expression level of sporopollenin biosynthesis-related genes which results in excessive sporopollenin accumulation, leading to thicker nexine (the inner layer of the exine) formation [41].

These studies indicated that normal sporopollenin biosynthesis might be required for proper pollen exine formation. In agreement with this point, in our study, overexpression of *SiPKSA* induced male sterility in transgenic *Arabidopsis*. The pollen walls of *SiPKSA*-overexpressing plants were defective. The baculum of the pollen exine was loosely and disorderly arranged compared to the wild-type plants. The thickness of the exine increased significantly, suggesting an increase in sporopollenin biosynthesis that led to its excessive accumulation in transgenic *Arabidopsis* plants (Figs. 4 and 5). qRT-PCR analysis revealed that the expression of several sporopollenin biosynthesis-related genes was significantlyinfluenced by the overexpression of *SiPKSA* during anther development, suggesting there might be a feedback regulation (Fig. 6).

Many studies have demonstrated that sporopollenin is a complex biopolymer comprised mainly of fatty acids and phenolic and aliphatic compounds [7, 14]. With the identification of some genes related to sporopollenin synthesis, an increasing number of biochemical experiments in vitro have been conducted to explore their biochemical function. Studies have shown that both *AtPKSA* and the closely related *AtPKSB* can catalyze the condensation of long-chain fatty acids acyl-CoA and malonyl-CoA to produce α-pyrone products. Accordingly, *AtPKSA* is known as a multifunctional enzyme and is vital for the synthesis of both pollen exine fatty acids and phenolics [15, 16]. Rice *OsLAP6/OsPKS1* shares similar products of enzymatic reactions with *AtPKSA/LAP6* [27]. Similarly, *PpASCL*, a homolog of *AtPKSA/LAP6* in moss, encodes a sporophyte-specific enzyme that possess similar catalytic activity as *AtPKSA/LAP6* in vitro [26].

In this study, we found that overexpression of *SiPKSA* in transgenic *Arabidopsis* affected the contents of various long-chain fatty acids compared to wild-type plants. Moreover, the contents of several phenolic compounds were increased in the *SiPKSA*-overexpressing lines (Fig. 7), suggesting that the overexpression of *SiPKSA* altered fatty acid metabolism in anthers. Although previous chemical analysis suggested that the primary components of sporopollenin precursors are polyhydroxy long-chain or very long-chain fatty acids, in addition to oxidized aromatic derivatives, the natural chemical monomer composition of sporopollenin remains poorly defined due to technical limitations [22, 23]. In addition, the homeostasis of fatty acid metabolism is critical for pollen and pollen wall development [42]. These reports support the observed changes in the content of several long-chain fatty acids during anther development in our study. In addition, our results indicate a great perturbation of the homeostasis of fatty acid metabolism in developing anthers of *SiPKSA*-overexpressing plants compared to wild type plant.

### Normal expression of *PKSA* might be required for fertile pollen formation

Previous studies have shown that *pksa* mutants in both *Arabiodopsis* and rice cause defective pollen exine and induce male sterility [15–17]. In addition, in tobacco, interference with the expression of *NtPKSA* also caused disorganized pollen surface sculptures [27]. These studies indicated that mutation in the *PKSA* gene might lead to abnormal pollen formation. Herein, we found that *SiPKSA* was highly expressed in sterile sesame during the tetrad release and microspore development stages compared with fertile sesame (Fig. 2C-D). Overexpression of *SiPKSA* induced male sterility in transgenic *Arabidopsis* (Fig. 3). The pollen walls of *SiPKSA*-overexpressing plants were defective. The baculum of the pollen exine was loosely and disorderly arranged compared to the wild-type plants (Fig. 4). The thickness of the exine increased significantly (Fig. 5), suggesting an increase in sporopollenin biosynthesis that led to its excessive accumulation in transgenic *Arabidopsis* plants. Therefore, we deduced that normal expression and activity of *PKSA* might be essential for proper pollen development. Any defect in its expression may lead to abnormal pollen formation via imperfect exine development. Additional investigations are required to explore the intricate regulatory mechanisms of PKSAs in general.

## Conclusion

In this study, we cloned and characterized sesame polyketide synthase A (*SiPKSA*). Sequence analysis and tissue expression pattern analysis showed that *SiPKSA* is a polyketide synthase closely related to the development of sesame anthers. We found that high expression of *SiPKSA* is associated with male sterility through abnormal pollen and pollen wall development. Overexpression of *SiPKSA* in transgenic *Arabidopsis* confirmed that normal activity of PKSAs is required for vibrant pollen formation. The overexpression of *SiPKSA* altered fatty acid metabolism and significantly influenced the expression of sporopollenin biosynthesis-related genes in transgenic *Arabidopsis*. Thus, *SiPKSA* is a potential candidate gene for the genetic improvement of sesame seed yield through hybrid breeding. An in-depth examination of the molecular mechanisms involved in *SiPKSA* regulation during anther development is required to adequately exploit this valuable genetic resource.

## Materials and methods

### Plant materials and growth conditions

The sesame male-sterile mutant 95 ms-5A and its fertile segregant 95 ms-5B [34] were cultivated under natural conditions at the experimental station of the Oil Crops Research Institute of the Chinese Academy of Agricultural Sciences (OCRI-CASS, Wuhan, China). *Arabidopsis* ecotype Colombia (Col-0) was grown under standard growth conditions, the temperature was maintained at approximately 22 °C, the light intensity was 130 μE/m^2^s, and the photoperiod was 16 h light/8 h dark. All of the plant materials were provided by the OCRI-CASS. Study complied with local and national regulations for using plants.

### Cloning and sequence analysis

The ORF of *SiPKSA* was amplified with the specific gene primers SiPKSA-f (5′-ATGTCCAACATCATCATCAACAGC-3′) and SiPKSA-r (5′-TCAAAGACTCCTAAGAAGAATGCCT-3′) via PCR using fertile sesame anthers in the tetrad stage as the template. The physical and chemical properties of *SiPKSA* were analyzed using the EXPASY database (https://web.expasy.org/protparam/). The PLANTCARE and PLACE databases were used to analyze the cis-acting elements of *SiPKSA* [43, 44].

For multiple sequence alignment analysis, *SiPKSA* and three other *PKSAs* (*AtPKSA*, At1g02050; *OsPKSA*, NP_001064891; *BnPKSA*, XP_013701181) were aligned by the ClustalX program. For phylogenetic analysis, *SiPKSA* and selected *PKSs* from other species *(Arabidopsis thaliana*, At4g34850; *Brassica napus*, XP_013707052; *Helianthus annuus*, XP_022010182; *Glycine max,* XP_003537759, XP_003516799; *Populus trichocarpa*, XP_002302511; *Oryza sativa*, XP_015646301; *Zea mays*, PWZ54429; *Arachis hypogaea,* XP_015971059, XP_015952719; *Mesicago truncatula,* XP_024638419; *Brassica rapa,* XP_009108697) were subsequently used to construct a phylogenetic tree via MEGA-X with the neighbor-joining method as the default and 1000 bootstrap replicates [45]. All sequences were downloaded from the NCBI database.

### Vector construction and plant transformation

First, the ORF of *SiPKSA* was inserted into TSV-007S vector (Tsingke, China) and sequenced. Subsequently, through double enzyme digestion (restriction site: *NdeI* and *SmaI*), the fragment was ligated into PRI101-AN (Takara, Japan) to generate the overexpression vector (35S::SiPKSA) according to previously described methods [46, 47]. The expression vector was then introduced into *Arabidopsis thaliana* (Col-0) through *Agrobacterium tumefaciens* (strain GV3101) using the floral dip method [48]. Positive transgenic lines were screened on 1/2 MS solid medium containing 50 μg/mL kanamycin. *Arabidopsis* plants transformed with empty vector (EV) and wild-type *Arabidopsis* (WT) were used as the control.

### Plant morphology and pollen viability analysis

When the seedlings were eight weeks old, the phenotypes of the transgenic plants were photographed using a Nikon D90 digital camera. Pollen grains from wild-type *Arabidopsis,* wild-type *Arabidopsis* transformed with empty vector, and *SiPKSA*-overexpressing plants were placed on a glass slide, and 0.5% acetyl carmine reagent which was dropped on them and mixed thouroughly. After covering the slide for 30 min, the excess liquid was removed and observed under a microscope. Photographs were captured using an optical microscope (IX71, Olympus). All plump and darkly stained pollen grains were determined to be fertile, and the collapsed and unstained pollen grains were considered sterile.

### Ultrastructure observation

For the scanning electron microscopy (SEM) observation, anthers sampled from freshly dehisced anthers of WT, EV, and *SiPKSA*-overexpressing plants were dehydrated in 30, 50, 70, 80, 90, 95 and 100% ethanol series. The dehydrated samples were dried to the critical point in liquid CO_2_ and coated with gold particles. The images were acquired by a scanning electron microscope (VEGA3, Tescan).

For transmission electron microscopy (TEM) observation, anthers at different developmental stages collected from WT and *SiPKSA*-overexpressing plants were fixed in 2.5% glutaraldehyde at 4 °C overnight. Next, the samples were postfixed with 1% osmium tetroxide and subsequently dehydrated twice in 30, 50, 70, 80, 90, 95, and 100% ethanol for 15–20 min each time. After that, the samples were infiltrated with a mixture of acetone and epoxy resin in different proportions. The dehydrated samples were then embedded in epoxy resin and polymerized at 60 °C (incubator) for 48 h. Furthermore, they were sliced at a thickness of 80–100 nm by an ultrathin slicer (EMUC7, Leica) and stained with uranium-lead double staining solution. Images were captured using a transmission electron microscope (TecnaiG^2^20 TWIN). The thickness of the exine was measured using Nano measurer 1.2 software (http://emuch.net/html/201402/7022970.html). Three biological replicates were performed, and three microspores were measured for each replicate. The measurements were repeated ten times for each sample, following the method of Wu et al. [41].

### RNA isolation, RT-PCR and qRT-PCR analysis

Sesame roots, stems, leaves, flower buds, and capsules were collected for expression profile analysis of *SiPKSA* in sesame. To compare the expression difference of *SiPKSA* between sterile sesame plants (95 ms-5A) and the fertile plants (95 ms-5B), anthers at different developmental stages (tetrad stage, microspore development stage, and mature pollen stage) from sterile sesame plants (95 ms-5A) and the fertile plants (95 ms-5B) were sampled. Expression was normalized to *SiActin* (SIN_1009011) [49]. For expression analysis of sporopollenin biosynthesis-related genes in *Arabidopsis* anthers at different developmental stages (stages 6–8, 9–10, 11–12) from the wild type and *SiPKSA*-overexpressing plants were sampled. Expression was normalized to *AtActin7* (At5g09810) [50]. Total RNA was extracted from each sample using TRIzol reagent (Invitrogen, USA). First-strand cDNA was synthesized by SuperScript II reverse transcriptase (Vazyme Biotech, Nanjing, China). qRT-PCR was carried out using the Roche Light Cycler 480 system. The relative expression level was calculated using the 2^−△△Ct^ method [51]. The primers used in this study were listed in Table S1.

### Fatty acids and their derivates measurements

Anthers (0.2 g) at different developmental stages sampled from WT and the *SiPKSA*-overexpressing plants were extracted twice with 1 mL of chloroform for 2 h. Next, the mixed supernatant was re-extracted and dried with a nitrogen blower. Finally, 0.25 mL of 5% potassium hydroxide-methanol solution was added, and the mixture was incubated at 60 °C for 30 min and subsequently quenched with n-hexane. The final supernatant was analyzed by gas chromatography-mass spectrometry (GC-MS). The chromatographic conditions of GC-MS were as follows: inlet temperature 280 °C, split ratio 20:1, carrier gas: He, carrier gas flow rate: 1.0 ml/min, ion source temperature 230 °C, scanning mass range 35 ~ 800 M. The injection volume was 1 μL. The temperature rising program: The initial temperature was 50 °C and kept for 1 min; the temperature was increased to 200 °C gradually (5 °C/min); finally, the temperature wasincreased to 230 °C and held for 10 min.

### Statistical analysis

All experiments were performed with three biological replicates, and the presented values are the mean ± SD. Statistical significance was determined using Student’s t-test (*P < 0.05 is considred significantly different, **P < 0.01 is considred highly significantly different).

## Supplementary Information

**Additional file 1: Supplementary Table 1.** Primers used for RT-PCR and qRT-PCR. **Supplementary Table 2.** *SiPKSA* cis-acting elements potentially associated with the anther development. **Supplementary Table 3.** Real time data and statistical analysis. **Supplementary Figure 1.** Expression analysis of *AtPKSA* in WT, EV and transgenic Arabidopsis. **Supplementary Figure 2.** The uncropped gel of the RT-PCR result of *SiPKSA* expression in different tissues of sesame. **Supplementary Figure 3.** The uncropped gel of the RT-PCR result of the expression of *SiPKSA* in sesame fertile and sterile anthers at different developmental stages. **Supplementary Figure 4.** The uncropped gel of the RT-PCR result of *SiPKSA* in *SiPKSA*-overexpressing lines. **Supplementary Figure 5.** The uncropped gel of the RT-PCR result of the expression of *AtPKSA* in *SiPKSA*-overexpressing lines.

## Data Availability

All data generated or analyzed during this study are included in this published article and its supplementary information files.

## Abbreviations

ORF: Open Reading Frame
WT: Wild Type
EV: Transformed Plants with Empty Vector
OX: Overexpression Lines
RT-PCR: Reverse Transcription-Polymerase Chain Reaction
qRT-PCR: Quantitative Real-Time PCR
SEM: Scanning Electron Microscope
TEM: Transmission Electron Microscope
