# Supplementary material for: Overexpression of sesame polyketide synthase A leads to abnormal pollen development in Arabidopsis
Source: BMC Plant Biol. 2022 Apr 2;22:165. doi: 10.1186/s12870-022-03551-7 (PMC8976376; doi:10.1186/s12870-022-03551-7)
Supplement: Supplementary file 1 — Additional file 1: Supplementary Table 1. Primers used for RT-PCR and qRT-PCR. Supplementary Table 2. SiPKSA cis-acting elements potentially associated with the anther development. Supplementary Table 3. Real time data and statistical analysis. Supplementary Figure 1. Expression analysis of AtPKSA in WT, EV and transgenic Arabidopsis. Supplementary Figure 2. The uncropped gel of the RT-PCR result of SiPKSA expression in different tissues of sesame. Supplementary Figure 3. The uncropped gel of the RT-PCR result of the expression of SiPKSA in sesame fertile and sterile anthers at different developmental stages. Supplementary Figure 4. The uncropped gel of the RT-PCR result of SiPKSA in SiPKSA-overexpressing lines. Supplementary Figure 5. The uncropped gel of the RT-PCR result of the expression of AtPKSA in SiPKSA-overexpressing lines. [file 12870_2022_3551_MOESM1_ESM.docx]

**Supplementary Table 1.** Primers used for RT-PCR and qRT-PCR

| Primer | Forward 5’-3’ | Reverse 5’-3’ |
| --- | --- | --- |
| SiPKSA-RT | ATGTCCAACATCATCATCAACAGC | TCAAAGACTCCTAAGAATGCCT |
| AtPKSA-qRT | GCAAAGCTAGGCCTGAGGAA | GCTTTGTTTGGTGGCCGAAA |
| AtPKSA-RT | CGAGAATGGTGGTTTCAGAGGT | GGCATTGGAAGCCAGTTTAGG |
| AtMS2-qRT | CGGTTTCTTAGCTAAAGTACTGATT | CGCAGCTTCTTTGCTTTTGG |
| AtCYP704B1-qRT | AGCGCGGAAGCGACTAATAC | AACGGCAGGGTAGAGACGAA |
| AtTKPR1-qRT | GCTTCAGGCTTCTTGGCTTC | AATCAGCCTTCACCAACCGT |
| AtTKPR2-qRT | GGACCAAAACCAACAAGTACTC | CTTCCATAGCCAATACATGTGC |
| At-Actin7 | TGGCCGATGGTGAGGATATT | AACGGCCTGAATGGCAACAT |
| Si-Actin | TTTGAGCAGGAACTGGACACT | ACAACACTTCTGGACAACGGA |

**Supplementary Table 2.** *SiPKSA* cis-acting elements potentially associated with the anther development

| **Motif** | **Location** | **Sequence** | **Function** |
| --- | --- | --- | --- |
| AGAAA-motif | several | AGAAA | cis-acting element required for pollen specific transcription |
| CGTCA-motif | +73 | CGTCA | cis-acting regulatory element involved in the MeJA responsiveness |
| GATA-box | several | GATA | cis-acting element responsible for the tissue specific promoter activity |
| GTGA-motif | +313/+1332/+1776 | GTGA | cis-acting element in the promoter of late pollen |
| Q-element | -1438 | AGGTCA | cis-acting element in enhancing activity of pollen-specific gene |
| MYB | -955  +1937 | TAACCA  CAACCA | cis-acting element in regulation of phenylpropanoid biosynthetic |
| CAAT-box | several | CAAT  CAAAT | cis-acting element in promoter and enhancer regions |
| G-Box | -1237 | CACGTT | cis-acting element involved in light responsiveness |
| [ATCT-motif](http://bioinformatics.psb.ugent.be/webtools/plantcare/cgi-bin/show_site_info.htpl?QWhere=ID_of_Site%20like%20'AATCTAATCC'&StartAt=0&NbRecs=10) | +803 | AATCTAATCC | cis-acting element involved in light responsiveness |

**Supplementary Table 3. Real time data and statistical analysis**

**
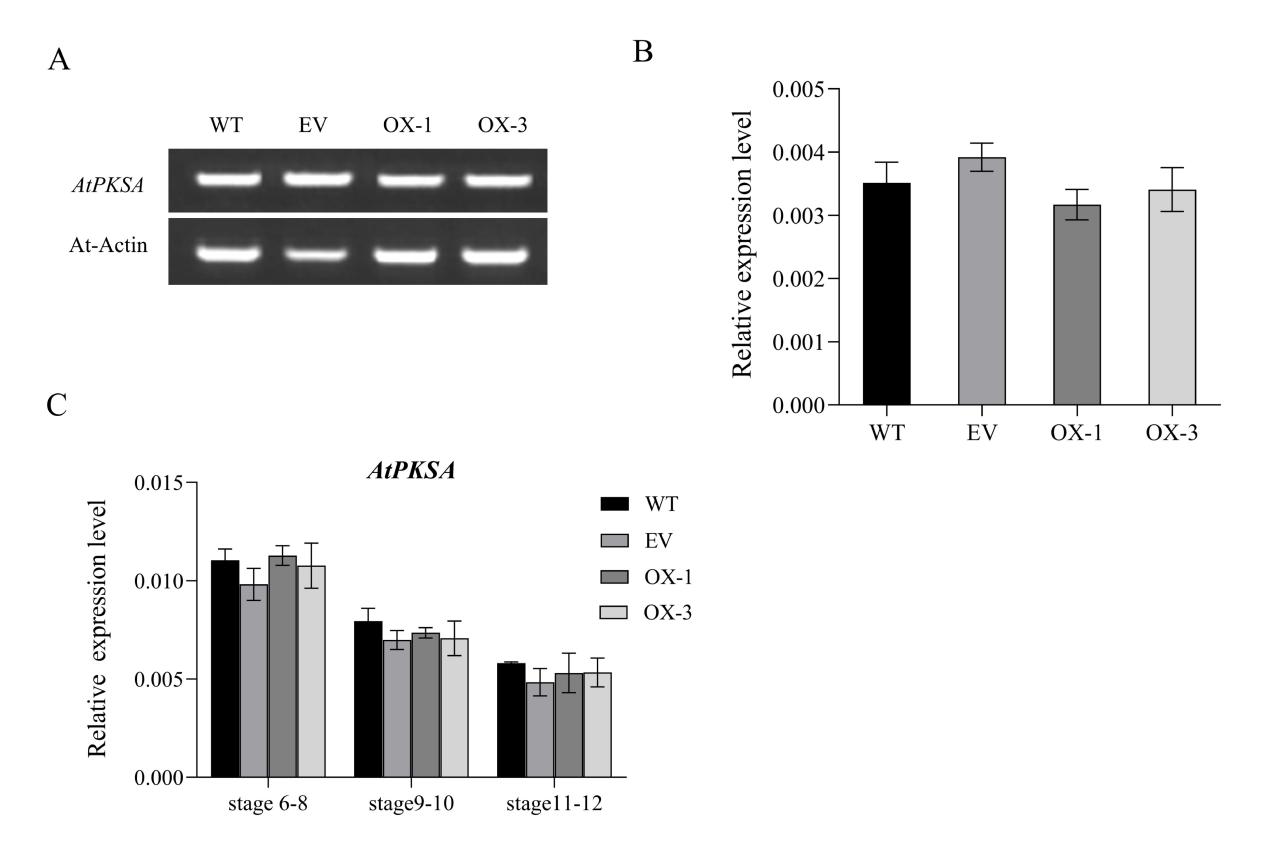
**

**Supplementary Figure 1.** Expression analysis of *AtPKSA* in WT, EV and transgenic Arabidopsis

(**A, B**) Expression analysis of *AtPKSA* in the WT, EV and *SiPKSA-*overexpressing lines (OX-1 and OX-3) by RT-PCR (A) and qRT-PCR (B). (**C**) Expression analysis of *AtPKSA* in the anthers of WT, EV and *SiPKSA*-overexpressing lines (OX-1 and OX-3) by qRT-PCR. *Actin* (At3g18780) was used as an internal standard to normalize the templates. The relative mRNA levels are represented as the mean ± SD (n = 3).


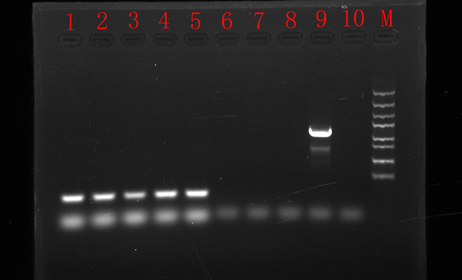


**Supplementary Figure 2.** The uncropped gel of the RT-PCR result of *SiPKSA* expression in different tissues of sesame. Bands 1-5 represented Root, stem, Leaf, Flower bud, Capsule, and the primer was SiActin. Bands 6-10 represented Root, stem, Leaf, Flower bud, Capsule, and the primer was SiPKSA-RT. M: DL2000 DNA Maker.


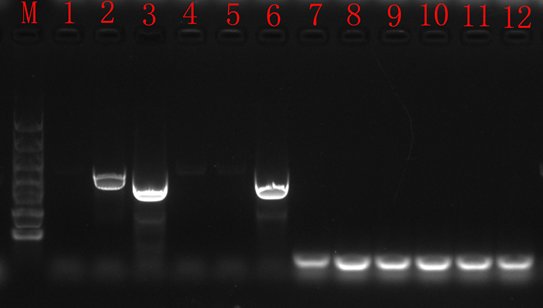


**Supplementary Figure 3.** The uncropped gel of the RT-PCR result of the expression of *SiPKSA* in sesame fertile and sterile anthers at different developmental stages. Bands 1-3 represented the tetrad stage, microspore-development stage, mature-pollen stage of sesame sterile anthers, respectively. Bands 4-6 represented the tetrad stage, microspore-development stage, mature-pollen stage of sesame fertile anthers, respectively. The primers with bands 1-6 and 7-12 were SiPKSA-RT and SiActin, respectively. M: DL2000 DNA Maker.


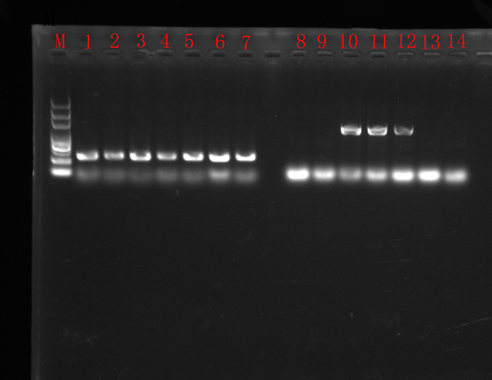


**Supplementary Figure 4.** The uncropped gel of the RT-PCR result of *SiPKSA* in *SiPKSA*-overexpressing lines. Bands 1-7 represented WT, EV, OX-1, OX-3, OX-2, WT, EV, respectively, and the primer was AtActin. Bands 8-14 represented WT, EV, OX-1, OX-3, OX-2, WT, EV, respectively, and the primer was SiPKSA-RT. M: DL2000 DNA Maker.


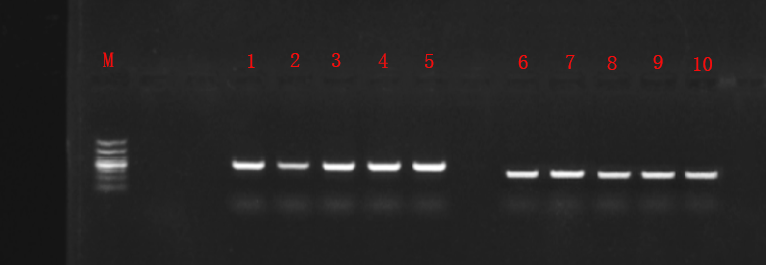


**Supplementary Figure 5.** The uncropped gel of the RT-PCR result of the expression of *AtPKSA* in *SiPKSA*-overexpressing lines. Bands 1-5 represented WT, EV, OX-1, OX-3, OX-2, respectively, and the primer was AtActin. Bands 6-10 represented WT, EV, OX-1, OX-3, OX-2, respectively, and the primer was AtPKSA-RT. M: DL1000 DNA Maker.
